# Isolation and analysis of a sake yeast mutant with phenylalanine accumulation

**DOI:** 10.1093/jimb/kuab085

**Published:** 2021-11-12

**Authors:** Akira Nishimura, Shota Isogai, Naoyuki Murakami, Natsuki Hotta, Atsushi Kotaka, Kengo Matsumura, Yoji Hata, Hiroki Ishida, Hiroshi Takagi

**Affiliations:** Graduate School of Science and Technology, Nara Institute of Science and Technology, 8916-5 Takayama, Ikoma, Nara 630-0192, Japan; Graduate School of Science and Technology, Nara Institute of Science and Technology, 8916-5 Takayama, Ikoma, Nara 630-0192, Japan; Research Institute, Gekkeikan Sake Co. Ltd., 101 Shimotoba-koyanagi-cho, Fushimi-ku, Kyoto 612-8385, Japan; Research Institute, Gekkeikan Sake Co. Ltd., 101 Shimotoba-koyanagi-cho, Fushimi-ku, Kyoto 612-8385, Japan; Research Institute, Gekkeikan Sake Co. Ltd., 101 Shimotoba-koyanagi-cho, Fushimi-ku, Kyoto 612-8385, Japan; Research Institute, Gekkeikan Sake Co. Ltd., 101 Shimotoba-koyanagi-cho, Fushimi-ku, Kyoto 612-8385, Japan; Research Institute, Gekkeikan Sake Co. Ltd., 101 Shimotoba-koyanagi-cho, Fushimi-ku, Kyoto 612-8385, Japan; Research Institute, Gekkeikan Sake Co. Ltd., 101 Shimotoba-koyanagi-cho, Fushimi-ku, Kyoto 612-8385, Japan; Graduate School of Science and Technology, Nara Institute of Science and Technology, 8916-5 Takayama, Ikoma, Nara 630-0192, Japan

**Keywords:** *ARO80*, Phenylalanine, 2-Phenylethanol, Sake yeast, Sake brewing

## Abstract

Sake is a traditional Japanese alcoholic beverage brewed by the yeast *Saccharomyces cerevisiae*. Since the consumption and connoisseurship of sake has spread around the world, the development of new sake yeast strains to meet the demand for unique sakes has been promoted. Phenylalanine is an essential amino acid that is used to produce proteins and important signaling molecules involved in feelings of pleasure. In addition, phenylalanine is a precursor of 2-phenylethanol, a high-value aromatic alcohol with a rose-like flavor. As such, adjusting the quantitative balance between phenylalanine and 2-phenylethanol may introduce value-added qualities to sake. Here, we isolated a sake yeast mutant (strain K9-F39) with phenylalanine accumulation and found a missense mutation on the *ARO80* gene encoding the His309Gln variant of the transcriptional activator Aro80p involved in the biosynthesis of 2-phenylethanol from phenylalanine. We speculated that mutation of *ARO80* would decrease transcriptional activity and suppress the phenylalanine catabolism, resulting in an increase of intracellular phenylalanine. Indeed, sake brewed with strain K9-F39 contained 60% increase in phenylalanine, but only 10% less 2-phenylethanol than sake brewed with the parent strain. Use of the *ARO80* mutant in sake brewing may be promising for the production of distinctive new sake varieties.

**One-Sentence Summary:**

The *ARO80* mutant is appropriate for controlling the content of phenylalanine and 2-phenylethanol.

## Introduction

Sake is a traditional Japanese alcoholic beverage made from polished and steamed rice by multiple parallel fermentations of the fungus *Aspergillus oryzae* and the yeast *Saccharomyces cerevisiae*, which produce saccharification enzymes and ethanol from glucose, respectively (Akaike et al., 2020). In the fermentation processes, yeast cells produce not only ethanol but also various metabolites, such as higher esters, higher alcohols, and organic acids, which define the characteristics of sake taste and flavor (Tatsukami et al., 2018). For example, 2-phenylethanol with a rose-like flavor, ethyl caproate with an apple-like flavor, and isoamyl acetate with a banana-like flavor are the major aroma components in sake. Due to growing interest in sake worldwide, there is a search for sake yeast strains that will result in unique sake with diversity of taste and flavor. Modification of metabolic pathways through the introduction of mutation(s) is a promising approach for construction of desirable sake yeast strains. Since 2019 in Japan, genome-editing technologies, such as CRISPR-Cas9 and TALENs, have been applied to breeding of crops and microbes for making foods and beverages, the same as conventional mutagenesis with chemicals or ultraviolet (Tsuda et al., 2019). In order to apply genome-editing technology, the removal of foreign DNAs (or RNAs) derived from the plasmid to introduce Cas9 and gRNA must be proven. However, to date, the Japanese government has not clarified how to prove the removal of foreign DNA or the criteria for removal. Therefore, the use of genome-editing technologies is currently difficult for construction of desirable sake yeast strains. Conventional mutagenesis would be the best method for breeding yeast strains.

Phenylalanine is a multifunctional amino acid in organisms. In addition to being a proteogenic amino acid, phenylalanine is used to produce important signaling molecules such as dopamine and epinephrine via tyrosine biosynthesis (Franco et al., 2021; Strandwitz, 2018). These molecules are involved in the performance of normal physiological functions in mammals, including mood and stress responses (Steckl & Ray, 2018). Additionally, phenylalanine is a precursor of 2-phenylethanol, which is a high-value aromatic alcohol with a rose-like flavor (Hazelwood et al., 2008; Stark et al., 2002). 2-Phenylethanol significantly contributes to the flavor and aroma of sake, beer, bread, cheese, and other fermented foods and has been widely used in the cosmetics and food industries (Chung et al., 2000; Stark et al., 2002). Adjusting the quantitative balance of phenylalanine and 2-phenylethanol in sake may introduce value-added qualities to sake.

**Fig. 1:**
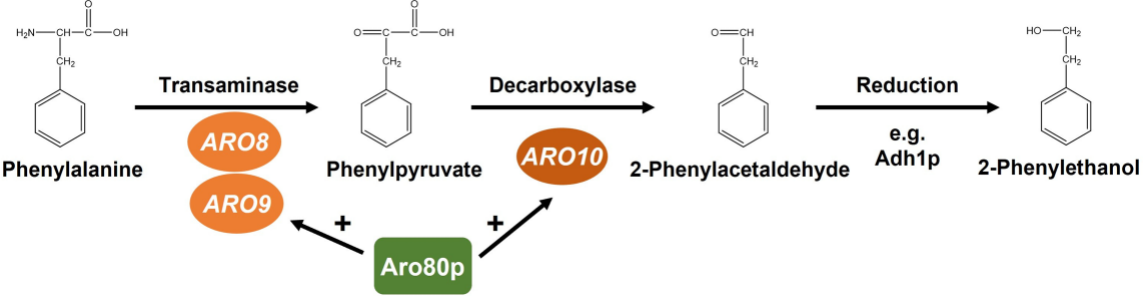
The Ehrlich pathway in *S accharomyces cerevisiae*. Phenylalanine is converted into 2-phenylethanol in the Ehrlich pathway. The biosynthetic pathway of 2-phenylethanol consists of three steps: Phenylalanine is deaminated to phenylpyruvate by transaminases (encoded by *ARO8* and *ARO9*). Next, phenylpyruvate is decarboxylated and converted into 2-phenylacetaldehyde by decarboxylase (encoded by *ARO10*). Finally, alcohol dehydrogenases (e.g., Adh1p) reduces 2-phenylacetaldehyde to 2-phenylethanol. The genes encoding enzymes that catalyze each step are indicated. The transcriptional activator Aro80p induces the transcription of *ARO9* and *ARO10. ARO8* is constitutively expressed and not affected by Aro80p.

2-Phenylethanol is synthesized from phenylalanine via the Ehrlich pathway (Fig. 1). The Ehrlich pathway consists of three steps: conversion of phenylalanine to phenylpyruvate by aromatic transaminase, decarboxylation of phenylpyruvate to phenylacetaldehyde by phenylpyruvate decarboxylase, and finally, reduction of phenylpyruvate to 2-phenylethanol by alcohol dehydrogenase (Hazelwood et al., 2008; Qian et al., 2019). Two isoenzymes are involved in the first step, transaminase I and II, which are encoded by the *ARO8* and *ARO9* gene, respectively. The enzyme in the second step is phenylpyruvate decarboxylase, which is encoded by the *ARO10* gene. It is known that both *ARO9* and *ARO10* are induced by aromatic amino acids (phenylalanine, tryptophan, or tyrosine), while *ARO8* is constitutively expressed (Iraqui et al., 1998). Thus, the expression levels of *ARO9* and *ARO10* greatly affect the amount of phenylalanine and 2-phenylethanol in yeast cells (Kim et al., 2014; Yin et al., 2015). The transcription of *ARO9* and *ARO10* is mainly regulated by Aro80p, a member of the Zn_2_Cys_6_ family of transcriptional activator proteins (Iraqui et al., 1999). The *ARO9* and *ARO10* promoters contain Aro80p-binding sites consisting of four CCG repeats separated by 7 bp (MacIsaac et al., 2006). Interestingly, Aro80p constitutively binds to the CCG motifs, and the binding status is not affected by intracellular aromatic amino acid levels (Lee & Hahn, 2013). To date, the mechanism by which aromatic amino acids regulate Aro80p has not been clarified.

In this study, we isolated a diploid sake yeast mutant that produced a higher phenylalanine level than that of its parent strain by conventional mutagenesis. This mutant had a missense mutation on the *ARO80* gene encoding the His309Gln variant of the transcriptional activator Aro80p involved in the biosynthesis of 2-phenylethanol from phenylalanine. The *ARO80* mutation caused a decrease in the transcriptional activity and a suppression of phenylalanine catabolism in yeast cells. Importantly, sake brewed with this mutant contained 60% increase in phenylalanine, but only 10% less 2-phenylethanol than sake brewed with the parent strain. The *ARO80* mutant used in this study may have promise for the production of distinctive new sakes.

## Materials and methods

### Strains and plasmids

Yeast strains used in this study are summarized in Table S1. The diploid Japanese sake yeast strain Kyokai no. 9 (K9-WT) and haploid laboratory yeast strain X2180-1A (wild-type [WT]) were used in this study.

The DNA sequence coding the triple hemagglutinin (3×HA)-tagged Aro80p, including 1,000 bp upstream and downstream of the open reading frame of *ARO80*, was purchased from GeneArt (Thermo Fisher Scientific). The DNA fragment was introduced to the *Eco*RI/*Sal*I-digested pYC130 containing the G418 resistance gene (supplied by National Research Institute of Brewing) via In-Fusion technology (Takara Bio) with primers, ARO80 pYC130 EcoRI Fw and ARO80 pYC130 SalI Rv (Table S2). The constructed plasmid was named as pYC130-Aro80-HA WT. A plasmid pYC130-Aro80-HA H309Q used for the expression of Aro80p H309Q mutant was constructed by the Quikchange method (Agilent, Santa Clara) with ARO80 H309Q Fw and ARO80 H309Q Rv (Table S2) and pYC130-Aro80 WT as a template. A map of the plasmids pYC130-Aro80-HA WT and pYC130-Aro80-HA H309Q is shown in Fig. S1. The plasmids pYC130-Aro80-HA WT and pYC130-Aro80-HA H309Q were introduced into yeast cells by the lithium acetate-PEG method (Gietz & Schiestl, 2007).

### Construction of the *ARO80*-disrupted strain

To construct an *ARO80*-disrupted strain (*aro80*Δ), an integration cassette containing a hygromycin-resistant gene was amplified by PCR with primers (ARO80 deletion Fw and ARO80 deletion Rv, Table S2) and pFA6a-hphMX6 (purchased from the AddGene repository) (Janke et al., 2004). The PCR fragments were integrated into the genome in strain X2180-1A by transformation. The correct integration event was verified by PCR using chromosomal DNA.

### Culture media

For culture of yeast cells, the following media were used; a nutrient-rich yeast extract–peptone–dextrose (YPD) medium (1% yeast extract, 2% peptone, and 2% glucose), and a synthetic medium (SD + Alt) containing 0.17% yeast nitrogen base without amino acids and ammonium sulfate, 0.5% allantoin, and 2% glucose.

### Selection of *p*-fluoro-dl-phenylalanine-resistant sake yeast mutants

Strain K9-WT was grown at 30ºC in YPD medium to the stationary growth phase and then treated with 5% ethyl methanesulfonate (EMS) in phosphate buffer (pH 7.0). After 60 min, 10% sodium thiosulfate was added to stop the mutagenesis reaction. The cells were collected, washed twice with sterile water, and plated on SD + Alt containing 100 μg/ml *p*-fluoro-dl-phenylalanine (PFP). After 3 days at 30°C, about 200 colonies were obtained, and after replating them on the PFP-containing medium, we finally selected 45 PFP-resistant mutants. The survival rate during mutagenesis was in the range of 20–40%.

### Spot test

Yeast cells were grown at 30°C in YPD medium to the stationary growth phase and diluted to 1.0 of optical density at 600 nm (OD_600_) with water. Aliquots (3 μl) of 10-fold serial dilutions were spotted on SD + Alt in the absence or presence of PFP. The plates were then incubated at 30°C for 3 days. When necessary, 50 μg/ml G418 was added to maintain the expression plasmids in yeast cells.

### Quantification of intracellular phenylalanine content

Yeast cells were inoculated into YPD medium starting from 0.1 of OD_600_. After incubation at 30°C for 24 h with shaking (250 rpm), cells (equaling 40 OD_600_ units) were collected, resuspended with 1.0 ml of water, and subsequently boiled for 20 min to release amino acids from cells. After centrifugation (5 min at 15,000 × g), phenylalanine content in the supernatant was determined with an amino acid analyzer (JLC-500/V, JEOL) (Nishimura et al., 2020).

### Whole-genome sequencing

Yeast strains were grown in YPD medium at 30°C for 1 day with shaking. The cells were then harvested and washed twice with sterile water. Genomic DNA was extracted by using Dr. GenTLE (from Yeast) High Recovery kit (Takara Bio). Libraries for sequencing analysis were prepared using the NEB Next Ultra DNA Library Prep Kit (New England Biolabs), and 18 618 686 reads with paired-end short reads of 150 bp were obtained using Illumina NovaSeq 6000 (Illumina) at about 100-fold coverage. For Adapter contamination and low-quality bases in sequence reads were removed by using Trimmomatic (v.0.38) software. The sake yeast Kyokai no. 7 genome (NRIB_SYGD, txid721032) as a reference was obtained from the Sake Yeast Genome Database (https://nribf1.nrib.go.jp/SYGD/, ver. 1.0). Bwa (v.0.7.17-r1188) was used for mapping the reads to the reference genome and subsequently Gatk (v.3.8.1) was used to extract mutation candidates. Finally, SnpEff (v.4.3t) was used for identifying mutation patterns and annotation. The sequencing processes were performed via a commercial DNA sequence service (Rhelixa).

### Bioinformatic analysis

Protein domains of Aro80p were predicted by the NCBI's conserved domain database (Marchler-Bauer et al., 2015). Multiple sequence alignments of Aro80p and the Aro80p homolog of other fungal species were performed using ClustalW (Larkin et al., 2007).

### Quantitative PCR analysis

Yeast cells were inoculated into YPD + G418 (250 μg/ml) medium starting from 0.1 of OD_600_. After incubation at 30°C for 24 h with shaking (250 rpm), cells were disrupted by using the Multi-Beads Shocker (Yasui Kikai) with 0.5-mm glass beads, and total RNA was extracted with the NucleoSpin RNA Plus kit (Takara Bio) according to the manufacturer's instructions. cDNA was synthesized from total RNA with the PrimeScript RT reagent Kit (Takara Bio). The relative abundance of *ARO8, ARO9*, and *ARO10* mRNAs was quantified by means of quantitative PCR with the Light Cycler 96 system (Roche) and SsoAdvanced Universal SYBR Green Supermix (Bio-Rad Laboratories). The following primer sets (listed in Table S2) were used in this analysis: ARO8 qPCR Fw and ARO8 qPCR Rv, PCR efficiency: 94.6%; ARO9 qPCR Fw and ARO9 qPCR Rv, PCR efficiency: 92.1%; ARO10 qPCR Fw and ARO10 qPCR Rv, PCR efficiency: 97.1%. The following PCR protocol was used: 95°C for 4 min followed by 40 cycles of denaturation at 95°C for 15 s and annealing/extension at 60°C for 30 s. Each gene's cycle threshold was normalized to a housekeeping gene *ACT1* and relative expression levels were calculated using the 2^−ΔΔ^*^CT^* method (Livak & Schmittgen, 2001).

### Chromatin immunoprecipitation assay

We performed the chromatin immunoprecipitation (ChIP) assay according to the previous reports (Lee & Hahn, 2013; Nishimura et al., 2019) with some minor modifications. Strain *aro80*Δ harboring plasmids pYC130-Aro80-HA WT or pYC130-Aro80-HA H309Q was inoculated into YPD + G418 (250 μg/ml) medium starting from 0.1 of OD_600_. After incubation at 30°C for 24 h with shaking (250 rpm), 1% formaldehyde was directly added to the medium. The samples were incubated for 2 h at 30°C and then treated with 350 mM glycine to stop the reaction. The cells were suspended with lysis buffer (50 mM Tris-HCl, 150 mM NaCl, 1% Triton X-100, and 0.1% Sodium deoxycholate; pH 8.0) and disrupted by using the Multi-Beads Shocker (Yasui Kikai) with 0.5-mm glass beads. The sample was ultrasonicated to prepare 100–2,000 bp DNA and incubated with anti-HA Magnetic Beads (Thermo Fisher Scientific) overnight at 4°C. DNA–Aro80p–antibody complexes were washed 5 times in lysis buffer, and eluted from the anti-HA Magnetic Beads by the incubation with elution buffer (50 mM Tris-HCl, 10 mM EDTA, and 1% SDS; pH 8.0) for 1 h at 70°C. To remove any contaminating RNAs, samples were treated with 5 μg/ml RNase A (Takara Bio) for 1 h at 37°C. Crosslinking between DNA and Aro80p was reversed by the degradation of proteins with 500 μg/ml proteinase K (Thermo Fisher Scientific) for 14 h at 65°C. DNA samples were purified with NucleoSpin Gel and PCR Clean-up (Takara Bio) and analyzed by PCR with primer sets (listed in Table S2).

### Small-scale sake brewing test

Sake yeast strains were cultivated in YPD medium at 30°C for 1 day. The cells were harvested by centrifugation and resuspended with water. The sake brewing test was carried out at 15°C. Sake mash consisted of 36 g of pre-gelatinized rice with a polishing ratio of 45%, 9 g of dry koji, 1.2 × 10^9^ yeast cells, 20 μl of 90% (vol/vol) lactic acid, and 89 ml of water. The fermentation profile was monitored by measuring the volume of evolved CO_2_ using Fermograph II (Atto). After fermentation, the sake mash was centrifuged. The general components of the resulting sake were analyzed by standard methods established by the National Tax Administration Agency (Murakami et al., 2020).

### Determination of 2-phenylethanol and 2-phenylethyl acetate

2-Phenylethanol and 2-phenylethyl acetate in the sake was quantified using headspace gas chromatography (GC) (Tsukatani et al., 2003). The determination of 2-phenylethanol and 2-phenylethyl acetate was determined by using GC-2010 plus (Shimadzu) with a TurboMatrix HS (PerkinElmer), a flame ionization detector and Stabilwax column (30 m × 0.53 mm, 50 μm film thickness) (GL Science). The chromatographic conditions were: column temperature 50°C (5 min), 50–100°C (5°C/min), 100–220°C (10°C/min), 220°C (3 min), injector temperature 220°C, detector temperature 220°C, carrier gas nitrogen (0.2 kPa), flow rate 1.0 ml/min.

### Statistical analysis

Data are presented as means ± standard deviation (SD) and statistical significance was evaluated using Student *t*-test or one-way/two-way analysis of variance (ANOVA) with Tukey's test for multiple group comparisons. These analyses were performed using Prism 7 (GraphPad Software). *p* < .05 was considered statistically significant.

## Results and Discussion

### Isolation of a sake yeast mutant with phenylalanine accumulation

**Fig. 2:**
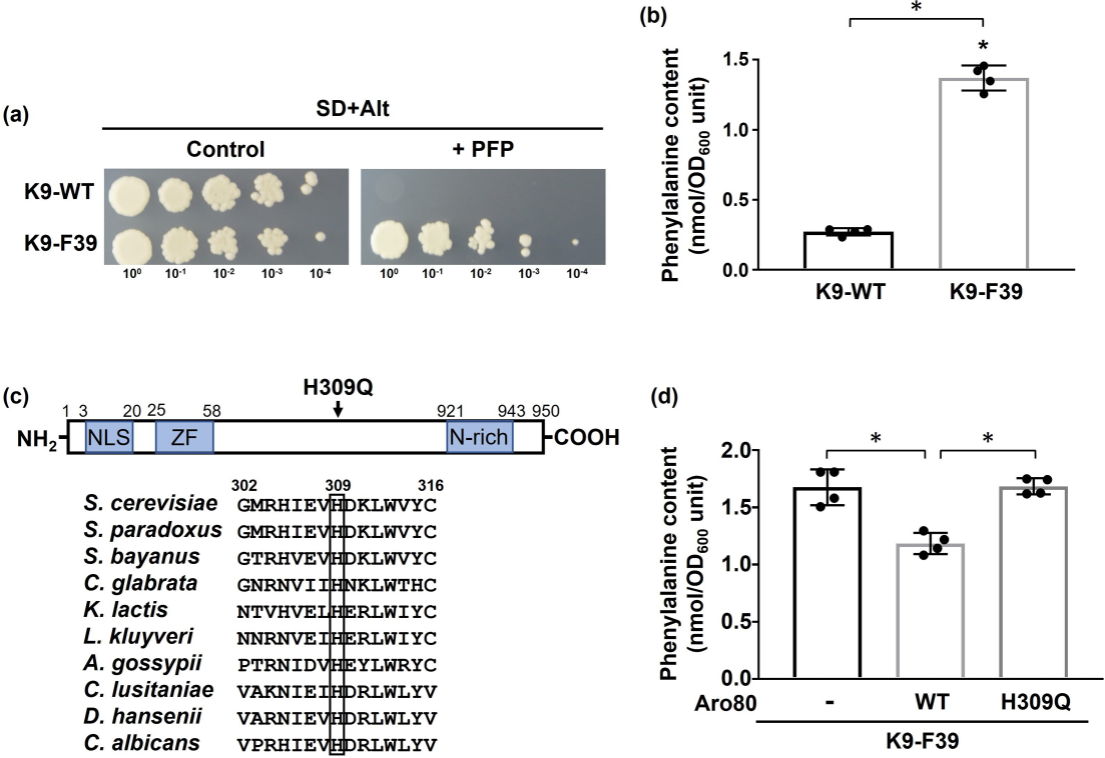
Isolation of a sake yeast mutant with phenylalanine accumulation. (a) Growth of strains K9-WT and K9-F39. Yeast cells from each strain were spotted onto SD + Alt agar plates in the absence and presence (+ PFP) of *p*-fluoro-dl-phenylalanine (100 μg/ml). The plates were incubated at 30°C for 2–3 days. (b) Intracellular phenylalanine. Strains K9-WT and K9-F39 were grown in YPD medium and intracellular phenylalanine was determined. Data are presented as means ± SD and statistical significance was determined by Student *t*-test. *p < .05. (c) Features of Aro80p. Upper panel shows multiple domains and a site of amino acid change (His309Gln) in strain K9-F39. A nuclear localization signal (NLS), the zinc finger domain (ZF), and the asparagine-rich domain (N-rich) are shown. Lower panel indicates the sequence alignment of the Aro80p proteins from *Saccharomyces cerevisiae* (*S. cerevisiae*), *Saccharomyces paradoxus* (*S. paradoxus*), *Saccharomyces bayanus* (*S. bayanus*), *Candida glabrata* (*C. glabrata*), *Kluyveromyces lactis* (*K. lactis*), *Lachancea kluyveri* (*L. kluyveri*), *Ashbya gossypii* (*A. gossypii*), *Candida lusitaniae* (*C. lusitaniae*), *Debaryomyces hansenii* (*D. hansenii*), and *Candida albicans* (*C. albicans*). Residues are numbered according to *S. cerevisiae* Aro80p and His309 is highlighted in a black box. (d) Intracellular phenylalanine. Strains K9-F39 harboring the empty vector (-), the wild-type (WT) and the H309Q variant Aro80p were grown in YPD medium and intracellular phenylalanine was determined. The *ARO80* genes were expressed under the original promoter. Data are presented as means ± SD and statistical significance was determined by one-way ANOVA with Tukey's test. *p < .05.

With the goal of discovering a new yeast that would produce distinctive sake with altered amounts of phenylalanine and 2-phenylethanol, we used the phenylalanine toxic analog, PFP, for screening of phenylalanine-accumulating mutants. PFP can compete with phenylalanine for incorporation into nascent proteins, resulting in cell death (Furter, 1998). Thus, the phenylalanine-accumulating cells are known to be resistant to PFP. By conventional mutagenesis with EMS, 45 PFP-resistant mutants were finally selected from the diploid sake yeast strain Kyokai no. 9 (K9-WT). In this study, we further analyzed one, which is referred to strain K9-F39, of these mutants, (Fig. 2a). We next measured the intracellular phenylalanine content in both K9-WT and the mutant K9-F39. As we expected, the intracellular level of phenylalanine in K9-F39 was much higher than that in K9-WT (Fig. 2b). To identify the genes responsible for phenylalanine accumulation in K9-F39, we performed whole-genome sequence analysis of K9-F39. We discovered 291 mutations with an amino acid substitution in the genome of K9-F39 in comparison with that of K9-WT. Among the mutated genes, we found *ARO80* as a gene related to phenylalanine metabolism. Sequencing results revealed that K9-F39 has a nucleotide G at position 927 in the *ARO80* gene encoding a transcriptional activator involved in phenylalanine catabolism whereas K9-WT has a nucleotide C at the same position. This mutation of C to G leads to the amino acid replacement of histidine to glutamine at position 309 (H309Q, Fig. 2c), showing that K9-F39 has a homozygous missense mutant of *ARO80*.

As shown in Fig. 1, Aro80p activates the expression of both *ARO9* and *ARO10*, which are involved in 2-phenylethanol biosynthesis from phenylalanine via the Ehrlich pathway (Iraqui et al., 1999). Thus, we hypothesized that this mutation confers a loss-of-function to Aro80p, leading to the suppression of the Ehrlich pathway in yeast cells expressing the H309Q variant of Aro80p. Homology analysis of the Aro80 proteins (950 amino acids) indicated that His at position 309 is fully conserved among the Aro80p homolog of other fungal species, implying the importance of His at position 309 (Fig. 2c). *In silico* analysis revealed the presence of a nuclear localization signal (NLS) and a zinc finger domain (ZF) on the amino-terminus in Aro80p, showing the DNA-binding ability of Aro80p. An asparagine-rich domain (N-rich) was found on the carboxyl-terminus in Aro80p, but its function is still unknown. The location of the amino acid replacement (His at position 309) is on the middle part of Aro80p, and there seem to be no conserved domains near the center of Aro80p. Therefore, it may be involved in the regulation of transcriptional activity in the presence of aromatic amino acids such as phenylalanine. We then introduced *ARO80*^WT^ or *ARO80*^H309Q^ into K9-F39. Fig. 2d shows that the expression of *ARO80*^WT^ caused a significant decrease in the intracellular phenylalanine content of K9-F39. However, there were no clear differences in the intracellular phenylalanine content between yeast cells harboring the empty vector and those expressing H309Q-Aro80p. These results suggest that the amino acid replacement of His to Gln at position 309 results in a loss-of-function of Aro80p activity.

### Characterization of the H309Q variant Aro80p

**Fig. 3:**
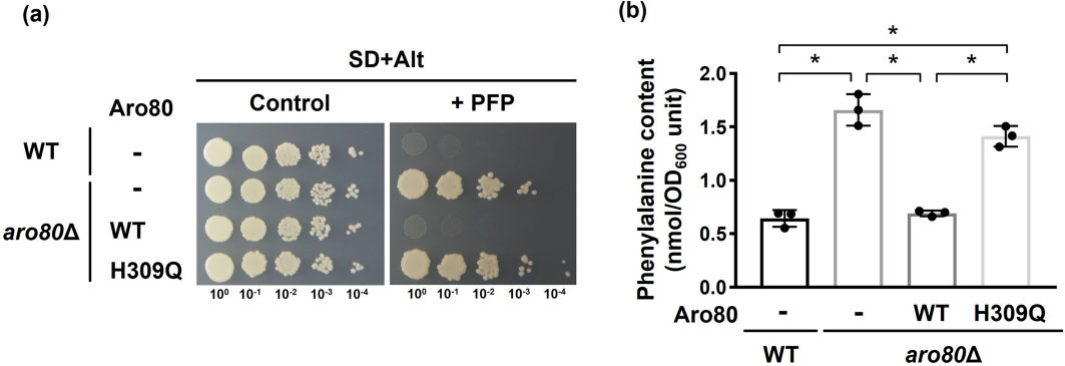
Characterization of the H309Q variant. (a) Growth of strains X2180-1A (WT) and its *aro80* disruptant (*aro80*Δ). Yeast cells expressing the wild-type (WT) and the H309Q variant Aro80p were spotted onto SD + Alt agar plates in the absence and presence (+ PFP) of *p*-fluoro**-**dl-phenylalanine (100 μg/ml). The plates were incubated at 30°C for 2–3 days. Minus indicates a negative control strain with the empty vector. (b) Intracellular phenylalanine. Strains WT harboring the empty vector (-), *aro80*Δ harboring the WT and the H309Q variant Aro80p were grown in YPD medium and intracellular phenylalanine was determined. The *ARO80* genes were expressed under the original promoter. Data are presented as means ± SD and statistical significance was determined by one-way ANOVA with Tukey's test. *p < .05.

To confirm the effect of the H309Q substitution on the accumulation of phenylalanine, we constructed an *ARO80*-deleted strain (*aro80*Δ) from the laboratory strain X2180-1a (WT) and expressed *ARO80*^WT^ or *ARO80*^H309Q^ in *aro80*Δ. As shown in Fig. 3a, *aro80*Δ cells showed a higher tolerance to PFP than WT cells. Additionally, the expression of the WT Aro80p conferred the sensitivity to PFP to *aro80*Δ cells, almost similar to WT cells, while *aro80*Δ cells expressing the H309Q variant were tolerant of PFP. We further measured the intracellular phenylalanine content in WT and *aro80*Δ cells (Fig. 3b). It was shown that the phenylalanine content in *aro80*Δ cells harboring the empty vector was significantly higher than that in WT cells. More importantly, the expression of the WT Aro80p in *aro80*Δ cells reduced the phenylalanine content to almost the same level as WT cells. By contrast, the expression of the H309Q variant had little effect on the intracellular phenylalanine level (*p* = .09, vs. *aro80*Δ harboring the empty vector). These results showed that the protein function of Aro80p was lost in the H309Q variant, suggesting that the Ehrlich pathway is suppressed in mutant *S. cerevisiae* cells expressing the Aro80p variant.

**Fig. 4:**
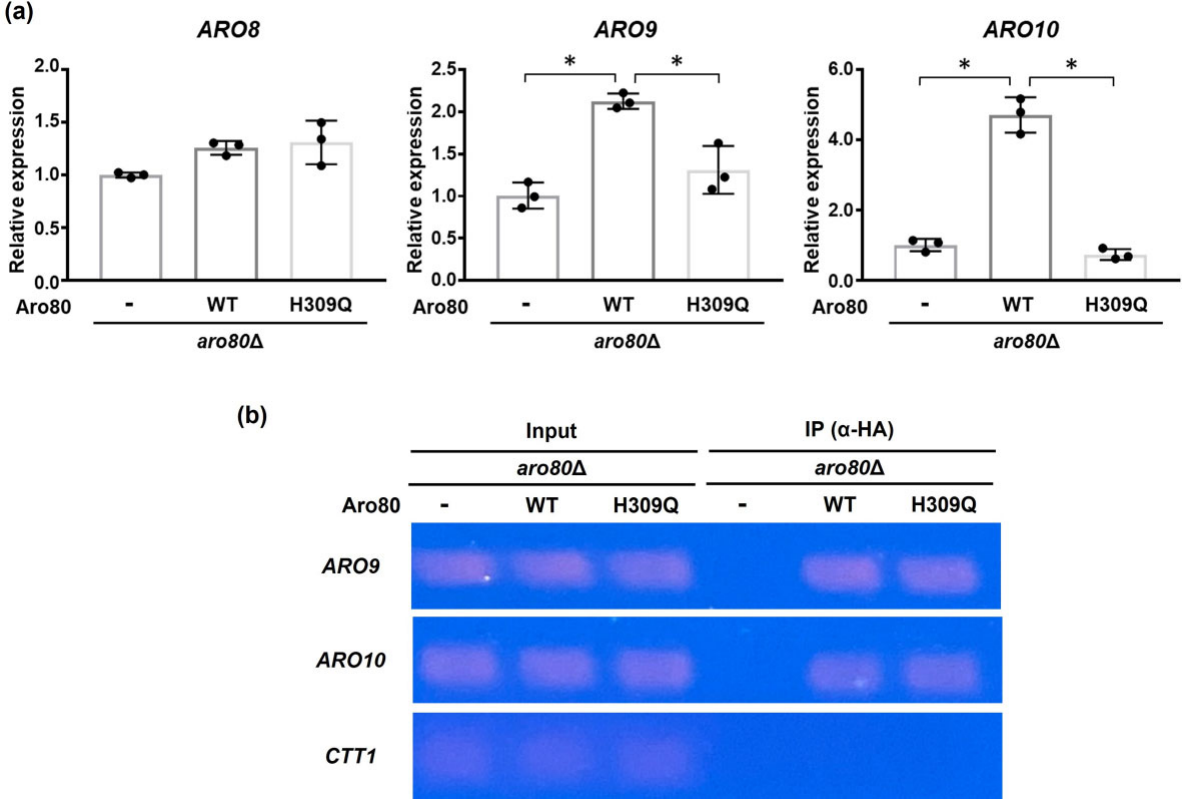
Transcriptional activity of the H309Q variant. (a) Transcription level of *ARO8, ARO9, ARO10* in strains *aro80*Δ harboring the empty vector (-), the wild-type (WT), and the H309Q variant Aro80p. Data are presented as means ± SD and statistical significance was determined by one-way ANOVA with Tukey's test. *p < .05. (b) The chromatin immunoprecipitation (ChIP) assay with the *ARO9* and *ARO10* promoters. Binding of the WT and the H309Q variant Aro80p to the promoter of *ARO9* and *ARO10* was determined using the ChIP assay with HA antibody. Input represents PCR results before immunoprecipitation. The *CTT1* promoter was used as a negative control.

Therefore, we determined the expression of genes (*ARO8, ARO9*, and *ARO10*) involved in the Ehrlich pathway in *aro80*Δ cells grown in rich medium (Fig. 4a). It was shown that the WT Aro80p had increased expression of both *ARO9* and *ARO10* in *aro80*Δ cells, but the H309Q variant did not contribute to the induction of these genes. The expression of *ARO8*, which is unregulated by Aro80p, was not affected by the introduction of the WT and H309Q variant Aro80p. Interestingly, the ChIP assay revealed that both the WT and H309Q variant Aro80p can interact with the promoter of *ARO9* and *ARO10* in the rich medium (Fig. 4b). These results indicate that the H309Q variant is not defective in binding activity to DNA related to the promoter of *ARO9* and *ARO10*, although the H309Q variant could not induce *ARO9* or *ARO10*. Lee and Hahn (2013) previously reported that Aro80p is constitutively bound to promoters even in the absence of inducers such as phenylalanine, suggesting that Aro80p is regulated by mechanisms other than DNA-binding capacity. One hypothesis is that the DNA-binding form of Aro80p can directly bind with phenylalanine, resulting in conformational changes of Aro80p. The conformational changes might correlate with transcriptional activity toward *ARO9* and *ARO10*. Such an activation mechanism has been reported in Put3, which is a well-studied transcriptional activator for the proline utilization pathway. It was shown that Put3 constitutively binds to DNA in a proline-independent manner (Sellick & Reece, 2005). Therefore, transcriptional activation by Put3 cannot be regulated by the control of its DNA-binding ability. It was suggested that proline can directly bind to the DNA-binding Put3, inducing its conformational change (Des Etages et al., 2001). This conformational change might result in unmasking the activation domain, thereby enabling the recruitment of the transcriptional machinery. Although the position and structure of the phenylalanine-binding site within Aro80p are still unknown, it is unlikely that the H309Q variant of Aro80p can interact with phenylalanine. In other words, the amino acid residue at position 309 within Aro80p may be involved in the phenylalanine-mediated regulation or the conformational change after binding to phenylalanine. Further analysis with the H309Q variant will be needed to reveal the regulatory mechanism of the Aro80p activity. In addition, Lee and Hahn (2013) reported that Gln3p and Gat1p, which are transcriptional activators of the nitrogen catabolite repression-sensitive genes, regulate the genes (*ARO9* and *ARO10*) involved in the Ehrlich pathway. In the presence of poor nitrogen sources, Gln3p and Gat1p are located in the nucleus, binding to the GATAA motifs within the promoter of *ARO9* and *ARO10* to activate transcription. Thus, the expression of *ARO9* and *ARO10* are synergistically regulated by Aro80p and Gln3p/Gat1p under the growth conditions containing poor nitrogen sources. Since cells grown in the nutrient-rich YPD medium were used in this study, Gln3p and Gat1p may be unfunctional. Therefore, Gln3p and Gat1p are unlikely responsible for the high phenylalanine content (Fig. 3b) and low expression (Fig. 4a) of *ARO9* and *ARO10* observed in the *ARO80* mutant. However, since the details of the interaction between Aro80p and Gln3p/Gat1p are largely unknown, further analyses will be necessary.

### Properties of sake brewed with a sake yeast mutant with phenylalanine accumulation

**Fig. 5:**
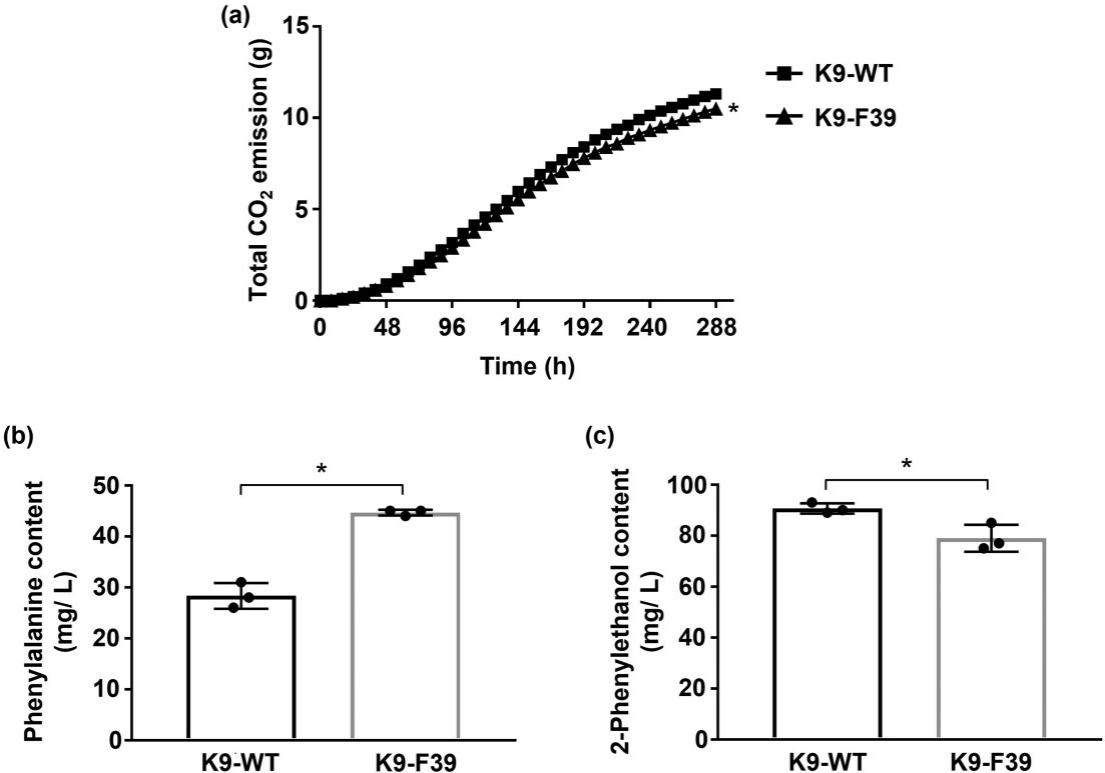
Small-scale sake brewing test. (a) Time course of CO_2_ production in sake brewed with strains K9-WT and K9-F39. Sake brewing was carried out at 15°C for 12 days. Data are presented as means ± SD and statistical significance was determined by two-way ANOVA with Tukey's test. *p < .05. (b) Phenylalanine content in sake brewed with strains K9-WT and K9-F39. Data are presented as means ± SD and statistical significance was determined by Student *t*-test. *p < .05. (c) 2-Phenylethanol content in sake brewed with strains K9-WT and K9-F39. Data are presented as means ± SD and statistical significance was determined by Student *t*-test. *p < .05.

We finally conducted a small-scale fermentation test to evaluate the characteristics of K9-F39 and properties of sake brewed with K9-F39. Total CO_2_ emission as an indicator of fermentation ability of yeast cells was monitored using the fermograph system (Fig. 5a). No differences in CO_2_ emission were observed in the early stages of sake brewing. However, in the late stages, the fermentation ability of K9-F39 was slightly lower than that of K9-WT. Similarly, the ethanol content in sake brewed with K9-F39 was less than that in sake brewed with K9-WT (Table S3). There was slightly more glucose remained in sake brewed with K9-F39 than with K9-WT. There was no significant difference in acidity or amino acidity between sakes brewed with the two strains. More importantly, sake brewed with K9-F39 strain contained 60% increase in phenylalanine, but only 10% less 2-phenylethanol than sake brewed with K9-WT. We next measured 2-phenylethyl acetate, ester of 2-phenylethanol, in the sake, since 2-phenylethyl acetate is an important flavoring agent with floral and rose-like odors same as 2-phenylethanol. But there was no significant difference in the content of 2-phenylethyl acetate in sake brewed with K9-WT (7.4 μg/ml) and K9-F39 (6.6 μg/ml). Metabolites (phenylpyruvate and phenylacetaldehyde) of the Ehrlich pathway other than 2-phenylethanol may be changed in strain K9-F39. The details will be clarified by metabolomics in the future. These results indicated that the use of the *ARO80* mutants is appropriate for controlling the content of phenylalanine and 2-phenylethanol. Sake brewing with the *ARO80* mutants could be promising for the production of a distinctive sake. In fact, there would seem to be unlimited possibilities of brewing with the *ARO80* mutants. We believe that not only sake yeast but also other brewing yeasts (e.g., those used to make beer, wine, and shochu) with the *ARO80* mutation could contribute to qualitative, value-added enhancement of these alcoholic beverages.

## Supplementary Material

kuab085_Supplemental_File

## Data Availability

The data underlying this article will be shared on reasonable request to the corresponding author.
