# Supplementary material for: Isolation and analysis of a sake yeast mutant with phenylalanine accumulation
Source: J Ind Microbiol Biotechnol. 2021 Nov 12;49(3):kuab085. doi: 10.1093/jimb/kuab085 (PMC9142190; doi:10.1093/jimb/kuab085)
Supplement: kuab085_Supplemental_File [file kuab085_supplemental_file.docx]

**Supplementary material**

**Isolation and analysis of a sake yeast mutant with phenylalanine accumulation**

**Akira Nishimura^1#^, Shota Isogai^1#^, Naoyuki Murakami^2^, Natsuki Hotta^2^, Atsushi Kotaka^2^, Kengo Matsumura^2^, Yoji Hata^2^, Hiroki Ishida^2^, and Hiroshi Takagi^1^***

^1^Graduate School of Science and Technology, Nara Institute of Science and Technology, 8916-5 Takayama, Ikoma, Nara 630-0192, Japan

^2^Research Institute, Gekkeikan Sake Co. Ltd., 101 Shimotoba-koyanagi-cho, Fushimi-ku, Kyoto 612-8385, Japan

*^#^* These authors contributed equally to this work.

*Corresponding author. Email: hiro@bs.naist.jp, Tel: +81-743-72-5420, Fax: +81-743-72-5429.

One-Sentence Summary: The *ARO80* mutant is appropriate for controlling the content of phenylalanine and 2-phenylethanol.

**This file includes:**

**Fig. S1** Map of pYC130-Aro80-HA

**Table S1** Yeas strains used in this study.

**Table S2** Primers used in this study.

**Table S3** Fermentation profiles

**
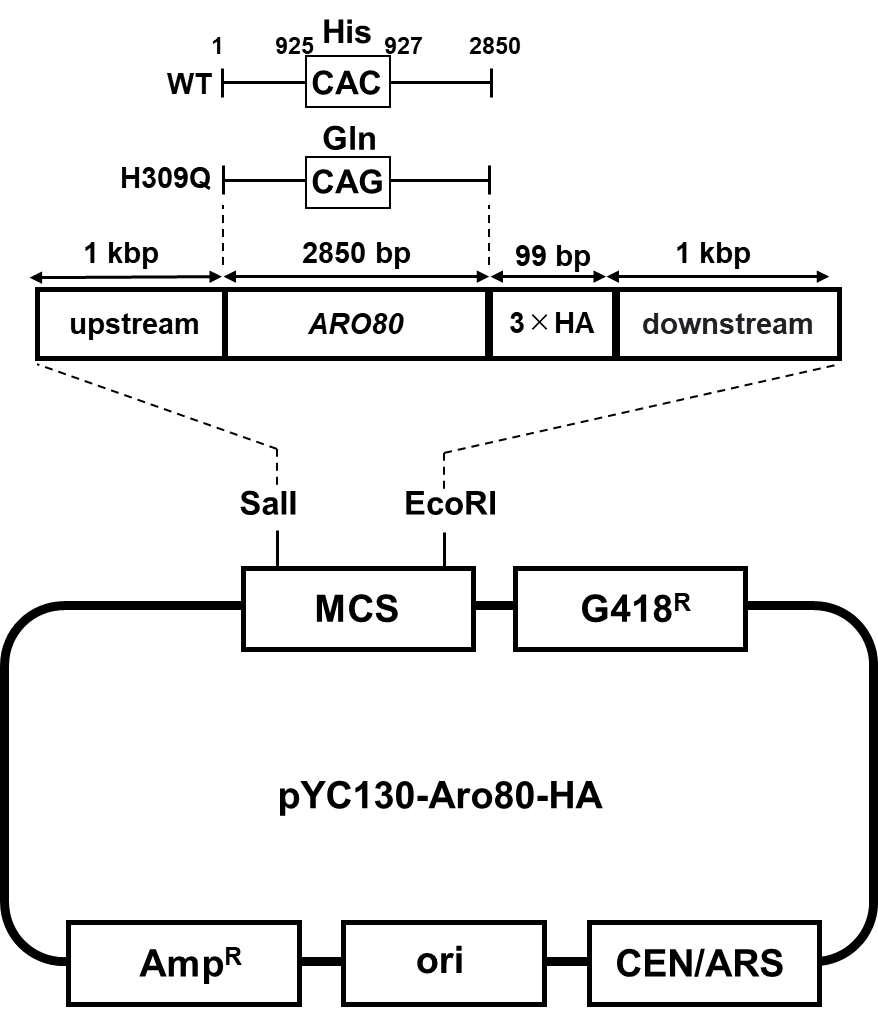
**

**Fig. S1** Map of pYC130-Aro80-HA. The multiple cloning site (MCS), an origin of replication for propagation in yeast (CEN/ARS), the G418 resistance gene (G418^R^), a marker of selection and an origin of replication for bacteria (ori and ampicillin resistance: Amp^R^) are indicated.
